# Determinants and patterns of service utilization and recourse to professionals for mental health reasons

**DOI:** 10.1186/1472-6963-14-161

**Published:** 2014-04-08

**Authors:** Marie-Josée Fleury, Guy Grenier, Jean-Marie Bamvita, Jean Caron

**Affiliations:** 1 Department of Psychiatry, McGill University, Douglas Hospital Research Centre, 6875 LaSalle Blvd, Montreal, Quebec H4H 1R3, Canada; 2 Douglas Hospital Research Centre, Montreal, Quebec H4H 1R3, Canada

**Keywords:** Service utilization, Mental health, Determinants, Professionals

## Abstract

**Background:**

This study has a dual purpose: 1) identify determinants of healthcare service utilization for mental health reasons (MHR) in a Canadian (Montreal) catchment area; 2) determine the patterns of recourse to healthcare professionals in terms of frequency of visits and type of professionals consulted, and as it relates to the most prevalent mental disorders (MD) and psychological distress.

**Methods:**

Data was collected from a random sample of 1,823 individuals interviewed after a two-year follow-up period. A regression analysis was performed to identify variables associated with service utilization and complementary analyses were carried out to better understand participants’ patterns of healthcare service utilization in relation to the most prevalent MD.

**Results:**

Among 243 individuals diagnosed with a MD in the 12 months preceding an interview, 113 (46.5%) reported having used healthcare services for MHR. Determinants of service utilization were emotional and legal problems, number of MD, higher personal income, lower quality of life, inability of individuals to influence events occurring in their neighborhood, female gender and, marginally, lack of alcohol dependence in the past 12 months. Emotional problems were the most significant determinant of healthcare service utilization. Frequent visits with healthcare professionals were more likely associated with major depression and number of MD with or without dependence to alcohol or drugs. People suffering from major depression, psychological distress and social phobia were more likely to consult different professionals, while individuals with panic disorders relied on their family physician only. Concerning social phobia, panic disorders and psychological distress, more frequent visits with professionals did not translate into involvement of a higher number of professionals or vice-versa.

**Conclusions:**

This study demonstrates the impact of emotional problems, neighborhood characteristics and legal problems in healthcare service utilization for MHR. Interventions based on inter-professional collaboration could be prioritized to increase the ability of healthcare services to take care especially of individuals suffering from social phobia, panic disorders and psychological distress. Others actions that could be prioritized are training of family physicians in the treatment of MD, use of psychiatric consultants, internet outreach, and reimbursement of psychological consultations for individuals with low income.

## Background

Epidemiological studies reveal that only a minority of individuals suffering from mental disorders use healthcare services [1-6]. For example, only 26% of Europeans suffering from a mental disorder in a 12-month period had consulted a professional according to a meta-analysis [7]. In Canada, the ratio of individuals using healthcare services for mental health reasons (MHR) was only 39% according to the 2002 Canadian Community Health Survey of Mental Health and Well-Being (CCHS 1.2) [8]. These findings underscore the need to find better ways to identify potential barriers to healthcare services.

Andersen’s behavioural model is the most common tool used for identifying determinants of healthcare service utilization [9]. In this model, variables are divided into predisposing, enabling and needs-related factors. Predisposing factors are individual characteristics that exist prior to the illness. Gender [10-12], age [11,13], marital status [4,14,15], and education are the chief predisposing factors associated with healthcare service utilization for MHR. Enabling factors are aspects that influence care delivery. The primary predisposing factors related to healthcare service utilization for MHR are income [16,17], insurance coverage [16,18], place of residence [19] and social support [20,21]. Needs-related factors include diagnosis, number and severity of mental disorders, as well as self-perception of mental and physical health [22,23]. According to the literature, needs are the strongest factors impacting healthcare service utilization [9,24,25]. Specific diagnoses tend to lead to much higher service utilization: schizophrenia [13], major depression [16], anxiety disorders [13] and co-occurring mental and substance-use disorders [24]. Duration of mental disorder [24], severity of symptoms [15], psychological distress [26] and poor physical health [23] are other needs-related factors that lead to higher service utilization.

Few studies have investigated determinants related to the types of professionals consulted [27-30]. According to a cross-sectional survey across six Europeans countries, people who are 49 or over and have less education and lower income tend to seek the services of their family physician rather than those of mental health providers [30]. Retired or unemployed individuals with less education and under psychiatric medication are more likely to consult psychiatrists only, while single persons tend to seek the services of other professionals only [29]. Studies have shown that individuals with major depression or mood disorders tend to rely on their family physician [30,31], while anxiety disorder sufferers are much more likely to call on psychiatrists and psychologists [32]. Persons with co-occurring mental health and substance-use disorders call on a broad range of professionals [31].

Previous studies have highlighted the key determinants of healthcare service utilization. Several variables, however, such as religious beliefs, neighborhood/geospatial information, dealings with the justice system, impulsivity, aggressive behaviour and violence have received little or no attention [33]. Few studies have compared the frequency of visits and type of professionals consulted in relation with various mental disorders. Do some diagnoses result in treatment being provided by one professional only or several types of professionals? If one seeks the services of a higher number of professionals, does that translate into more frequent visits?

This study has a dual purpose: 1) identify determinants of healthcare service utilization for mental health reasons in a Canadian (Montreal) catchment area; 2) determine the pattern of recourse to healthcare professionals in terms of frequency of visits and type of professionals consulted, and as it relates to the most prevalent mental disorders and psychological distress.

## Methods

### Study design and setting

This article is based on cross-sectional analyses from a broader longitudinal study in an epidemiological catchment area in Montreal, Canada’s second-largest city with a population of 3.6 million. The catchment area had a population of 269,720 spread over four neighborhoods ranging in population from 29,680 to 72,420. A third of the residents had low income (versus 23% in the province of Quebec and 35% in Montreal). In two neighbourhoods, the proportion of low income earners was close to half. Healthcare services are delivered primarily by a psychiatric hospital delivering specialized care (second- and third-line services) and two health and social service centers (created through the merger of a general hospital, community local service centers and nursing homes) offering both primary and specialized healthcare. Primary care is provided also by about 40 medical clinics and a similar number of private psychologists, along with 16 mental community-based agencies [34].

### Selection criteria and survey sample

To be included in the study, participants had to be aged between 15 and 65, reside in the catchment area, approve to participate after clear explanation of the study and fill in a consent form. For those aged 15 to 17, parents had to give authorization before the interview. Participants were selected so as to obtain a representative sample in terms of age, gender and socio-economic status, i.e. representative of varying degrees of educational attainment within the area, as well as geographically, i.e. recruiting participants from all areas of the territory proportionally to population density.

Data were prospectively collected from June 2007 to December 2010. At baseline (T1: June 2007 to December 2008), 2,434 individuals took part in the survey, for a co-operation rate of 48.7%. All participants were contacted for a second interview (T2) between June 2009 and December 2010. Only 611 were lost or excluded at follow-up because they had moved out of the catchment area or had died, and 1,823 responded to T2, for a retention rate of 74.9%. The attrition rate at T2 (25.1%) included only 138 refusals to participate (5.7%); 230 individuals (9.4%) had moved outside the catchment area, 231 (9.4%) were not reachable, and 12 (0.5%) had died. This attrition rate after two years was better than that observed in American epidemiological catchment areas after one year (20.4%, including 12.6% refusals) [35]. The attrition rates were higher among youths, singles, the less-educated, low income earners and those with substance dependence. This is similar to what was observed in other epidemiological catchment areas studies [35-37]. Douglas Mental health Institute Research Ethics Board Committee approved the research. The sampling strategy and data collection (especially at T1) are described in detail in related publications [33,34,38].

#### Variables and measurement instruments

The dependent variable was "healthcare utilization in the 12 months prior to the interview at T2". Independent variables were measured at T2 and organized according to Andersen’s behavioural model of healthcare service utilization, comprising predisposing determinants (age, gender, education, self-perception of physical health, self-perception of mental health, importance attributed to spirituality, number of children in the household, and problems with the law in past 12 months), enabling factors (household income, personal income, quality of life, neighbourhood characteristics, and social support) and needs-related factors (type and number of mental disorders, emotional problems, harm caused by violence, aggressive behavior [all of those in the past 12 months], and psychological distress). Table 1 displays the instruments used to measure the variables.

**Table 1:** Variables assessed in the study

**Variables**		**Measuring instruments**
**Predisposing factors**	Age	Canadian Community Health Survey of Mental Health and Well-Being CCHS 1.2 [39]
	Gender	
	Education	
	Self-perception of physical health	
	Self-perception of mental health	
	Importance attributed to spirituality	
	Number of children in the household	
	Problems with the law in past 12 months	
**Enabling factors**	Household income	CCHS 1.2 [39]
	Personal income	
	Quality of life	Satisfaction with Life Domains Scale [40]
	Neighborhood characteristics	Physical Conditions of the Neighbourhood [41]
		Neighbourhood Disorder Scale [42]
		Community Participation Scale [43]
	Social support	Resident Disempowerment Scale [44]
		Social Provisions Scale (SPS) [45]
**Needs-related factors**	Mental disorders (MD) in past 12 months (major depression, mania, panic disorder, social phobia, agoraphobia, post-traumatic stress disorder, alcohol dependence, drug dependence)	Composite International Diagnostic Interview (CIDI), [39]
	Number of MD in past 12 months	
	Emotional problems in past 12 months	CCHS 1.2 [39]
	Victims of violence in past 12 months	CCHS 1.2 [39]
	Aggressive behaviors in past 12 months	Modified Observed Aggression Scale (MOAS) for aggressive behaviours [46]
	Psychological distress	K-10 psychological distress scale [47]

### Analyses

Analyses entailed descriptive analyses (frequency distribution, mean values and standard deviation), bivariate analyses (comparison analyses using chi-square tests between participants who used healthcare services and those who did not), and multiple logistic regression. The regression analysis identified variables associated with service utilization for MHR at T2. The first step was to identify the variables that, based on the Andersen factors, had a significant bivariate relationship (p < 0.10) with service utilization. These variables were then entered into a multivariable logistic regression analysis. A two-tailed P < 0.05 was considered statistically significant for an independent association between the outcome and a given independent variable. The model goodness-of-fit was assessed using the Hosmer-Lemeshow test while the total variance explained by the model was calculated using Nagelkerke R^2^.

Two sets of complementary analyses were carried out to gain a better understanding of participants’ patterns of healthcare service utilization as they related to the most prevalent mental disorders. The first set, which considered participants who had used healthcare services at T2, entailed distribution of mean values along with standard deviations of frequency of visits with healthcare professionals in connection with major depression, panic disorder, social phobia, psychological distress and number of mental disorders, including or excluding dependence to alcohol and drugs. Comparison tests were carried out to determine those associations using the ANOVA t-test. The second set of analyses consisted in frequency distribution of visits with healthcare professionals at T2 in connection with major depression, panic disorder, social phobia and psychological distress. Comparison tests were carried out using Pearson’s chi-square test and Fisher’s exact test to assess the link between mental disorders and recourse to healthcare professionals.

## Results

From a total of 1,823 participants who responded to the questionnaire at T2, 243 individuals (13.3%) had at least one mental disorder and were selected for subsequent analyses. The proportion of females was twice that of males (Table 2). The majority (68%) reported having more than high-school education. The majority felt that their physical (55%) and mental health (54%) was excellent or very good. The most prevalent mental illness was major depression. Only 47% of individuals having mental disorders had used healthcare services for mental reasons in the 12 months before the interview.

**Table 2:** Descriptive characteristics of participants at baseline (T1) and comparison analyses according to healthcare service utilization (N = 243)

			**Total sample (N=243)**	**No utilization (n=130)**	**Utilization (n=113)**	**P value (chi-square test)**
			**[n/Mean(% SD)]**	**[n/Mean(% SD)]**	**[n/Mean(% SD)]**	
Predisposing factors	Age [Mean(SD)]		43.3 (12.8)	42.2 (13.6)	44.5 (11.8)	.165
	Gender [n(%)]	Female	160 (65.8)	73 (56.2)	87 (77.0)	.001
		Male	83 (34.2)	57 (43.8)	26 (23.0)	
	Education [n(%)]	High school or less	78 (32.1)	53 (40.8)	25 (22.1)	.002
		Beyond high school	165 (67.9)	77 (59.2)	88 (77.9)	
	Self-perception of physical health [n(%)]	Poor or fair	42 (17.3)	25 (19.2)	17 (15.0)	.073
		Good	68 (28.0)	42 (32.3)	26 (23.0)	
		Excellent or very good	133 (54.7)	63 (48.5)	70 (61.9)	
	Self-perception of mental health [n(%)]	Poor or fair	37 (15.2)	28 (21.5)	9 (8.0)	0.001
		Good	76 (31.3)	43 (33.1)	33 (29.2)	
		Excellent or very good	130 (53.5)	59 (45.4)	71 (62.8)	
	Importance attributed to spirituality [n(%)]		141 (58.0)	75 (57.7)	66 (58.4)	.910
	Number of children in the household [Mean(SD)]		0.5 (0.9)	0.5 (1.0)	0.5 (0.9)	.740
	Problems with the law in past 12 months		11 (4.5)	3 (2.3)	8 (7.1)	.090
Enabling factors	Household income [Mean(SD)]		49552.1 (41246.8)	46105.7 (32878.9)	53517.0 (49002.7)	.167
	Personal income [Mean(SD)]		28215.1 (21927.0)	24845.7 (17118.1)	32091.4 (25942.6)	.014
	Quality of life score [Mean(SD)]		95.2 (17.7)	97.4 (16.6)	92.7 (18.6)	.040
	Neighbourhood characteristics scores	Neighborhood physical condition [Mean(SD)]	43.6 (12.3)	42.5 (12.2)	44.8 (12.4)	.136
		Neighborhood disorder [Mean(SD)]	3.9 (1.4)	3.9 (1.4)	3.9 (1.4)	.921
		Community involvement [Mean(SD)]	0.8 (1.0)	0.7 (1.1)	0.9 (1.0)	.173
		Resident disempowerment [Mean(SD)]	12.8 (6.2)	13.6 (6.2)	11.9 (6.0)	.036
	Social support score [Mean(SD)]		77.8 (10.8)	78.3 (11.0)	77.2 (10.5)	.454
Needs	Mental disorders (MD) in past 12 months	Major depression [n(%)]	147 (60.5)	66 (50.8)	81 (71.7)	.001
		Mania [n(%)]	13 (5.3)	6 (4.6)	7 (6.2)	.587
		Panic disorder [n(%)]	17 (7.0)	7 (5.4)	10 (8.8)	.296
		Social phobia [n(%)]	41 (16.9)	21 (16.2)	20 (17.7)	.748
		Agoraphobia [n(%)]	7 (2.9)	1 (0.8)	6 (5.3)	.069
		Alcohol dependence [n(%)]	44 (18.1)	30 (23.1)	14 (12.4)	.033
		Drug dependence [n(%)]	28 (11.5)	18 (13.8)	10 (8.8)	.227
		Post-traumatic stress disorder [n(%)]	18 (7.4)	8 (6.2)	10 (8.8)	.426
	Number of MD in past 12 months [Mean(SD)]		1.3 (0.6)	1.2 (0.5)	1.4 (0.8)	.025
	Emotional problems in past 12 months [n(%)]		187 (77.0)	80 (61.5)	107 (94.7)	< .001
	Problems related to mental health in past 12 months [n(%)]		133 (54.7)	42 (32.3)	91 (80.5)	< .001
	Victim of violence in past 12 months [n(%)]		25 (10.3)	13 (10.0)	12 (10.6)	.874
	Aggressive behaviours in past 12 months [n(%)]		39 (16.0)	25 (19.2)	14 (12.4)	.150
	Psychological distress score [Mean(SD)]		15.3 (7.5)	14.4 (7.2)	16.5 (7.8)	.030

Bivariate analyses between those who used healthcare services for MHR and those who did not showed that the former were mostly females (Table 2). They were more educated and felt that their mental health was excellent or very good, although they had legal problems (predisposing factors). They had also on average a higher personal income (self-reported from the questionnaire of the CCHS 1.2, Table 1), a stronger perception of their inability to affect neighborhood conditions According to the Resident Disempowerment scale and a lower quality of life (enabling factors). They had more mental disorders and suffered mostly from major depression, alcohol dependence and, marginally, agoraphobia. They also had higher psychological distress (according to the K-10 psychological distress scale), and more emotional and mental-health-related problems (needs-related factors) (Table 2).

The variables associated with healthcare service utilization for MHR in bivariate analyses were used to create the multiple logistic regression model assessing variables independently linked to healthcare utilization displayed in Table 3. Eight variables were retained in this model. Half were positively associated (legal problems, emotional problems and number of mental disorders and personal income). The other half were negatively associated (gender, quality of life score, perceived inability to influence neighbourhood conditions and, marginally, alcohol dependence in the past 12 months). The two largest determinants of healthcare utilization were emotional problems (OR: 8.672) and legal problems (OR: 6.976). As shown by the Hosmer-Lemeshow Statistic, this model has an acceptable goodness-of-fit and explains 37% of the total variance.

**Table 3:** Variables independently associated with healthcare service utilization among participants with meantl disorders (MD) and/or psychological distress after 18 months follow-up: multiple logistic regression analysis (T2) (N = 243)

	**B**	**SE**	**Wald**	**df**	**P**	**OR**	**95% CI**	
							**LB**	**UB**
Gender (male)	-.945	.342	7.618	1	.006	.389	.199	.760
Legal problems in past 12 months	1.943	.902	4.633	1	.031	6.976	1.190	40.909
Alcohol dependence in past 12 past months	-.950	.488	3.797	1	.051	.387	.149	1.006
Emotional problems in past 12 months	2.160	.455	22.535	1	< .001	8.672	3.555	21.157
Number of mental disorders in past 12 months	.605	.273	4.900	1	.027	1.832	1.072	3.130
Personal income	.000	.000	8.411	1	.004	1.000	1.000	1.000
Quality of life score	-.023	.006	16.925	1	< .001	.978	.967	.988
Perceived inability to influence neighbourhood conditions	-.062	.024	6.912	1	.009	.940	.897	.984

A total of 467 participants (with or without a mental disorder diagnosis) visited healthcare professionals for MHR. Table 4 shows their pattern of recourse to healthcare professionals for the most prevalent mental disorders and psychological distress. Almost 30% of participants saw a family physician, psychiatrist, psychologist and other professionals (nurse, social worker, addiction counsellor, etc.) together, while 25% called on a family physician, a psychiatrist and a psychologist. When comparing participants with and without major depression, we found that the former were more likely to consult all types of healthcare professionals, alone or in combination. Compared to participants without psychological distress, those who did have this problem more often sought help from all types of health professionals, although they were unlikely to request the services of other professionals only. Compared to participants without social phobia, those who suffered from this anxiety disorder tended to see a psychologist or other professional only; a psychologist with a family physician or psychiatrist; a psychologist, family physician and psychiatrist combined, or all three along with another professional. Finally, participants with panic disorder (as compared to those without) tended to rely on their family physician only.

**Table 4:** Participants’ characteristics as regards type of professional in service utilization, most prevalent mental disorders (MD) and psychological distress in past 12 months as reported at T2 (N = 1,823)

		**Service utilization**			**Major depression**			**Panic disorder**			**Social phobia**			**Psychological distress**		
		**(n=467)**			**(n=157)**			**(n=33)**			**(n=61)**			**(n=651)**		
		**n**	**%**	**P value**	**n**	**%**	**P value**^**1**^	**n**	**%**	**P value**^**2**^	**n**	**%**	**P value**^**3**^	**n**	**%**	**P value**^**4**^
Professionals visited in past 12 months reported at T1	Family physician	70	15.0		27	17.2	< .001*	4	12.1	.035**	4	6.6	.292**	41	6.3	< .001*
	Psychologist	48	10.3		16	10.2	< .001*	3	9.1	.054**	5	8.2	.006*	28	4.3	.001*
	Psychiatrist	36	7.7		13	8.3	< .001*	1	3.0	.485**	3	4.9	.117**	21	3.2	.004*
	Other professionals	37	7.9		9	5.7	.001*	1	3.0	.495**	4	6.6	.033**	17	2.6	.189*
	Psychologist and family physician	102	21.8		32	20.4	< .001*	4	12.1	.109**	8	13.1	.009*	56	8.6	< .001*
	Psychiatrist and family physician	93	19.9		30	19.1	< .001*	4	12.1	.084**	6	9.8	.087*	52	8.0	< .001*
	Psychiatrist and psychologist	69	14.8		21	13.4	< .001*	3	9.1	.126**	7	11.5	.001*	38	5.8	.001*
	Psychiatrist, family physician and psychologist	116	24.8		32	20.4	< .001*	4	12.1	.154**	9	14.8	.006*	61	9.4	< .001*
	Psychiatrist, family physician, psychologist and other professionals	138	29.6		35	22.3	< .001*	4	12.1	.309**	11	18.0	.002*	71	10.9	< .001*

*Pearson’s chi-square test; **Fisher’s exact test.

^1^Compare to those without Major depression.

^2^Compare to those without Panic disorder.

^3^Compare to those without Social phobia.

^4^Compare to those without Psychological distress.

Table 5 displays the frequency of visits for each professional in connection with major depression, panic disorder, social phobia, psychological distress and number of mental disorders, including or excluding dependence to alcohol and drugs. Compared to participants without major depression, those who suffered from this illness had much more frequent visits with all types of professionals, except those classified as "other" (social worker, nurses, etc.) alone. Participants with a higher number of mental disorders, including or excluding dependence to alcohol and drugs, visited much more often with all types of professionals, except psychiatrists or other professionals alone. Compared to participants without panic disorder, those suffering from this condition had much more frequent visits with their family physician only, a psychologist only, a psychologist and family physician, a psychiatrist and family physician, a psychiatrist and psychologist or all three types of healthcare professionals together. Compared to participants without psychological distress, those with this problem had much more frequent visits with their family physician only or with a psychiatrist. Finally, there were no differences between participants with or without social phobia in terms of frequency of visits with healthcare professionals.

**Table 5:** Participants’ pattern of frequency of visits with professionals as regards most prevalent mental disorders (MD), psychological distress in past 12 months and number of MD as reported at T2 (N = 1823)

	**Major depression**			**Panic disorder**			**Social phobia**			**Psychological distress**			**Number of mental disorders in past 12 months excluding dependence to alcohol and drugs**			**Number of mental disorders in past 12 months including dependence to alcohol and drugs**		
	**(n=157)**			**(n=33)**			**(n=61)**			**(n=651)**								
**Frequency of visits to professionals**	**Mean**	**SD**	**P value**^1 ^*	**Mean**	**SD**	**P value**^2 ^*	**Mean**	**SD**	**P value**^3 ^*	**Mean**	**SD**	**P value**^4 ^*	**Mean**	**SD**	P value**	**Mean**	**SD**	P value**
Family physician	0.9	3.1	< .001	0.6	2.3	.002	0.7	3.6	.954	0.3	2.1	.028	0.6	0.8	< .001	0.7	1.0	< .001
Psychologist	1.8	9.5	.001	2.2	9.4	.004	0.9	3.2	.122	0.5	3.4	.082	0.6	1.0	.002	0.7	1.0	.011
Psychiatrist	0.2	0.9	.001	0.1	0.5	.512	0.1	0.6	.452	0.1	0.7	.138	0.7	1.0	.180	0.8	1.1	.438
Other professionals	0.8	4.3	.168	0.1	0.7	.531	0.8	3.3	.148	0.6	6.7	.947	0.5	0.9	.697	0.6	1.1	.580
Psychologist and family physician	2.7	10.5	< .001	2.8	11.6	.023	1.5	4.8	.258	0.8	4.4	.054	0.5	0.8	< .001	0.6	0.9	< .001
Psychiatrist and family physician	1.2	3.5	< .001	0.7	2.6	.013	0.8	3.7	.679	0.4	2.3	.041	0.6	0.8	< .001	0.6	0.9	< .001
Psychiatrist and psychologist	2.0	9.9	.001	2.3	9.5	.035	1.0	3.3	.073	0.6	3.6	.121	0.6	0.9	.002	0.6	1.0	.011
Psychiatrist, family physician and psychologist	3.0	11.0	< .001	2.9	11.8	.046	1.6	4.8	.236	0.9	4.6	.128	0.5	0.8	< .001	0.6	0.9	< .001
Psychiatrist, family physician, psychologist and other professionals	3.8	12.5	.001	3.1	12.0	.107	2.4	6.3	.143	1.5	8.3	.163	0.4	0.8	.028	0.5	0.9	.010

*Independent sample t-test; **ANOVA t-test.

^1^Compare to those without Major depression.

^2^Compare to those without Panic disorder.

^3^Compare to those without Social phobia.

^4^Compare to those without Psychological distress.

## Discussion

Our results revealed that almost half of participants (47%) with at least one mental disorder used healthcare services for MHR. This ratio is higher than that of previous studies. Usually the percentage of persons with a mental disorder seeking healthcare services hovers between 33 and 40 percent [8,48-50]. The proportion of individuals with mental disorders among our full sample (13.3%) was also higher than those found in the 2002 Canadian Community Health Survey of Mental Health and Well-Being (CCHS 1.2) for Canada (9.5%) and for all the provinces (between 6.7% and 11.3%) [8]. We can hypothesize that higher utilization of healthcare services for MHR (as well as prevalence of mental disorders) in our study results from the local presence of a psychiatric hospital and a large network of public and community-based organizations involved in mental health. It may be that frequent recourse to psychologists by participants drove up the rate of service utilization. In our study, as in the province of Quebec overall, psychologists were the second most consulted professionals after family physicians [4,8], while they rank third in other Canadian provinces, tied with psychiatrists and behind family physicians and social workers. The rate of Quebecers who consult psychologists is almost twice that of the Canadian average [8]. In 2008, the ratio of psychologists in Quebec was 104 per 100,000 population, as opposed to 48 per 100,000 for all of Canada [51].

The three major needs-related factors of healthcare service utilization are emotional problems, number of mental disorders and, marginally, lack of alcohol dependence. Emotional problems were the key determinant of healthcare service utilization for MHR. Such problems often involve interpersonal relationships [52]. Hence, past or recent experiences of crime and violence are a common cause of emotional problems [53,54]. A perceived inability to influence neighborhood conditions is another aspect that could explain the high prevalence of emotional problems. These findings contradict those of previous research, which found that individuals with emotional problems do not usually request healthcare services [55-57]. This difference could stem from the fact that, in our study, participants self-reported emotional problems, whereas these problems were linked to an established diagnosis such as depression or anxious disorder in other studies [58]. Various studies report that individuals with emotional problems seek professional help only when confronted with serious problems [59]. Some research found that individual behavior and beliefs (wishing to solve their problem by themselves, belief that emotional problems would go away) pose serious barriers to treatment for individuals with emotional problems [55-57]. Such attitudes tend to be more frequent among younger individuals with less education [55]. The literature, however, exposes other major obstacles to healthcare service utilization by this clientele, including cost of services, unawareness of available services, waiting times, transportation problems, bad experiences with professionals, doubts concerning the efficacy of treatment, and fear of stigmatization [22,55-57,59-61].

The link between the number of mental disorders over the previous 12 months and healthcare service utilization for MHR was reported previously [62]. Individuals with multiple mental disorders have more reasons to pursue treatment [2]. Substance dependents are not usually heavy service users, unless they suffer also from co-occurring mental or physical disorders [63,64].

Three enabling factors influenced healthcare service utilization: personal income, control over events occurring in the neighborhood, and quality of life. The link between healthcare service utilization and higher personal income has often been reported [11,18,65,66]. While individuals with low income are more likely to be affected by mental disorders [49], they use significantly fewer healthcare services. This under-utilization can be explained by some variables strongly associated with a disadvantaged socio-economic status [49]. Individuals with low income and suffering from mental disorders are usually unmarried [4,14,48,49] and have less education [4,6,14,15,31,48] and a weaker social network [20,23,31]. Some authors note that individuals with higher education are more apt to understand their problems and to know where to find help [6,15]. Moreover, it is easier for them to understand their needs, and they tend to be more receptive to treatment [13,15]. For their part, spouses and friends can help individuals to recognize their problems and encourage them to see a professional [31,33]. Cost of services, however, severely limits access to healthcare services, even in countries, such as Canada, that have a universal public healthcare system. [22]. For example, some professional services, such as the majority of those of psychologists, are not covered by public health insurance (in Quebec and in most western countries) and are only available, therefore, to persons with the financial means to afford them or able to rely on an insurance plan to cover the cost [16,67]. Reimbursement policies have a positive impact on service use [29]. In a previous paper dealing with T1 in the same catchment area, we found that 63% of individuals who consulted a psychologist had insurance covering psychological services [33].

The link between perceived inability to influence neighborhood conditions and healthcare service utilization can be explained by the deleterious effects of social disorganization on mental health [68]. Inability to change the social environment is a major cause of stress, which can trigger emotional problems, psychological distress, anxiety and depression or lead to substance abuse [68-71]. Conversely, social cohesion in the neighbourhood increases well-being and reduces daily stressors [72]. Moreover, residential stability increases interactions between neighbours and fosters stronger social relationships [71,73]. Concerning quality of life, some studies have found that social fears both decrease quality of life and increase healthcare service utilization [74-76]. Moreover, quality of life is lower among individuals suffering from multiple mental disorders [74], who are also more likely to use services.

Only two predisposing factors impacted healthcare service utilization: female gender and legal problems in the past 12 months. Concerning gender, several studies have found much higher healthcare service utilization for MHR among females than males [6,10-12,14,16,24,48,77,78]. A possible explanation is that females suffer more frequently from anxiety disorders, mood disorders and psychological distress [79]. Compared to the other gender, males are also less aware of their mental health [80-82], have more difficulty in accepting a diagnosis of mental illness [12] and tend to avoid seeking help until there is a sharp deterioration of their condition [6,48,78]. Physical symptoms are the determining factor of healthcare service utilization by males [83]. Moreover, it appears that family physicians are more comfortable treating females, so that males are more commonly referred to specialists [6]. We were surprised to find that legal problems in the past 12 months could be a key determinant of healthcare service utilization for MHR. Usually, conflicts with the law create a barrier to healthcare service utilization since prison inmates often fail to get help for their mental health problems [84,85]. Previous run-ins with the law may have led some participants with mental disorders to seek help, or it could be that individuals with legal problems have agreed to receive mental healthcare services in exchange for a reduced sentence [86].

In terms of type of professionals consulted and frequency of visits in connection with mental disorders and psychological distress, the study confirmed the expected link between major depression and recourse to different professionals and a high frequency of visits [16]. This mental disorder is the one for which the help of healthcare professionals is most often sought [16]. According to the literature, individuals with major depression benefit the most from interventions based on inter-professional collaboration [87-89]. The focus on major depression in anti-stigmatization campaigns and current mental health reforms could also explain the frequent recourse to healthcare professionals [90].

There is an apparent contradiction concerning the number of professionals consulted and the frequency of visits among individuals with psychological distress, panic disorder and social phobia. Participants with psychological distress are more likely to consult all types of professionals. However, in term of frequency of visits, a family physician alone or both a family physician and psychiatrist are the only professionals on whom they call significantly more often. Curiously, psychological distress had no significant impact on frequency of visits with a psychologist. Many individuals suffering from psychological distress may thus prefer medication to long-term psychotherapy. Moreover, the number of psychotherapy sessions allowed by private insurance is limited [91]. The study confirmed that individuals with panic disorder frequently consult healthcare professionals [32,92]. Suicide ideation or attempts are higher among sufferers of panic disorder, which explains their frequent use of emergency departments and ambulatory services [32,92]. As observed in other studies, however, the only professional usually called upon for panic disorder was the family physician [87-89], probably because related symptoms are generally physical (hyperventilation, heart palpitation, chest pain, etc.). It is possible that individuals with panic disorder seek the services of several professionals according to the severity of their condition but use their family doctor as a regular source of healthcare. Having a regular medical care provider is a positive factor in service utilization [13]. Conversely, individuals with social phobia call on several professionals but seem reluctant to commit to a regular healthcare provider, perhaps because their condition leads them to avoid social contacts and makes them reluctant to use healthcare services regularly [92].

On a final note, a greater number of mental disorders in the past 12 months, including or excluding dependence to alcohol or drugs, did not result in frequent visits to a psychiatrist or other professional alone. According to Meadows et al. [19], individuals who consult psychiatrists also tend to see other professionals. The frequency of visits by alcohol or drug dependents was no different from that of individuals with no dependence and this could indicate a lack of integrated treatment for co-occurring disorders [93]. Mental health professionals and addiction specialists usually work in isolation from each other, and this is reflected in the treatment of co-occurring disorders [94,95].

### Limitations

This study presents some limitations. First, the full spectrum of mental disorders was not included in our analysis. Individuals with personality disorders or serious mental disorders such as schizophrenia are reported to be heavy service users [96-98]. Second, we did not have information about the severity of symptoms. Previous studies reported greater healthcare service utilization among individuals with more severe psychiatric symptoms [15,64,99]. Third, our data cannot adequately address some determinants which can be serious barriers to healthcare service utilization, such as duration, waiting times, past experiences with professionals or satisfaction of the quality of care [22,29,61,99]. These situational aspects are just as important as financial considerations in cases of people not using healthcare services or dropping out of care [61]. Fourth, certain variables, such as homelessness, availability of private insurance and access to a family physician, that could influence service utilization for MHR, were not measured. Finally, the results could reflect the characteristics of the population of the catchment area and may not be generally applicable to other areas or populations.

## Conclusion

This study was innovative in that it looked specifically at healthcare service utilization by individuals with mental disorders, while previous studies had put greater emphasis on the general population. Moreover, this study is of interest in that it included several variables, such as neighbourhood characteristics and legal problems, which are not usually considered. To our knowledge, no other research has analyzed healthcare service utilization with a set of such comprehensive variables using Andersen’s behavioural model.

The results show that emotional problems are the chief determinant of healthcare service utilization for MHR among individuals with mental disorders. A key issue is to provide access to services for individuals suffering from emotional problems, which are often the result of interpersonal conflicts. Males are especially loath to speak of their emotional problems and to use healthcare services until confronted with a severe deterioration of their condition. Strategies to be considered include promoting help phone lines, crisis centers, bibliotherapy, internet outreach and self-help groups. This study also broke new ground by uncovering a link between legal problems in the past 12 months and healthcare service utilization among individuals with mental disorders. Problems with the law are usually considered a barrier to healthcare service utilization, although they may induce some individuals with mental disorders to seek help. Moreover, the study emphasizes the need to improve neighbourhood conditions to prevent mental disorders among poorer populations, and to increase outreach activities aimed at these groups, where there is a higher prevalence of mental disorders, psychological distress or emotional problems. Finally, this study contributes to the development of knowledge concerning frequency of visits and number of professionals consulted in connection with mental disorders and psychological disorders. Based on our analyses, major depression was the only diagnosis showing a correlation between frequency of visits and number of professionals consulted. Concerning social phobia, panic disorder and psychological distress, more frequent visits with professionals seem to have no bearing on the number of professionals consulted. Priority could be given to initiatives relying on inter-professional collaboration to increase the ability of healthcare services to take care of individuals suffering from social phobia, panic disorder and psychological distress. Better training of family physicians in mental health, use of psychiatric consultants, internet outreach, and free access to psychological services would be among the measures to be prioritized to increase integration and capacity of services to treat individuals affected by mental disorders or psychological distress and improve access to mental healthcare services.

## Competing interests

The authors declare that they have no competing interests.

## Authors’ contributions

JC designed the epidemiological catchment area study. MJF, GG and JMB designed the specific research. JMB performed the statistical analyses with the help of JC. MJF and GG wrote the article. All authors have read, revised and approved the final manuscript.

## Pre-publication history

The pre-publication history for this paper can be accessed here:

http://www.biomedcentral.com/1472-6963/14/161/prepub
